# Intramuscular Immunization with Chemokine-Adjuvanted Inactive Porcine Epidemic Diarrhea Virus Induces Substantial Protection in Pigs

**DOI:** 10.3390/vaccines8010102

**Published:** 2020-02-24

**Authors:** Fu-Chun Hsueh, Yen-Chen Chang, Chi-Fei Kao, Chin-Wei Hsu, Hui-Wen Chang

**Affiliations:** 1 Graduate Institute of Molecular and Comparative Pathobiology, School of Veterinary Medicine, National Taiwan University, Taipei 10617, Taiwan; jim820723@gmail.com (F.-C.H.); yenchenchang@ntu.edu.tw (Y.-C.C.); robby951159@gmail.com (C.-W.H.); 2 School of Veterinary Medicine, National Taiwan University, Taipei 10617, Taiwan; fei81005@gmail.com

**Keywords:** CC chemokine, adjuvants, porcine epidemic diarrhea virus, mucosal immunity

## Abstract

Intramuscular (IM) immunization is generally considered incapable of generating a protective mucosal immune response. In the swine industry, attempts to develop a safe and protective vaccine for controlling porcine epidemic diarrhea (PED) via an IM route of administration have been unsuccessful. In the present study, porcine chemokine ligand proteins CCL25, 27, and 28 were constructed and stably expressed in the mammalian expression system. IM co-administration of inactivated PEDV (iPEDV) particles with different CC chemokines and Freund’s adjuvants resulted in recruiting CCR9+ and/or CCR10+ inflammatory cells to the injection site, thereby inducing superior systemic PEDV specific IgG, fecal IgA, and viral neutralizing antibodies in pigs. Moreover, pigs immunized with iPEDV in combination with CCL25 and CCL28 elicited substantial protection against a virulent PEDV challenge. We show that the porcine CC chemokines could be novel adjuvants for developing IM vaccines for modulating mucosal immune responses against mucosal transmissible pathogens in pigs.

## 1. Introduction

Since late 2010, emerging highly virulent porcine epidemic diarrhea virus (PEDV) strains have resulted in severe economic losses in several counties in North America and Asia [1,2]. PEDV strains cause porcine epidemic diarrhea (PED), characterized by vomiting, severe diarrhea, and dehydration in seronegative pigs at all ages, with high mortality in suckling pigs [3]. Many attempts have been made to develop a safe and protective vaccine for controlling PED [4,5,6,7,8,9,10]. However, similar to studies on most enterotropic and mucosal transmissible viral diseases [11,12,13,14], using intramuscular (IM) administration of inactive or subunit vaccines combined with commercial adjuvants, such as multiple emulsions of water/oil/water or a B subunit of *Escherichia coli* heat-labile enterotoxin (LTB) induced production of systemic IgG and neutralizing antibodies but failed to elicit a mucosal IgA response and efficient protection [6,7,15,16,17]. Furthermore, poor efficacies of currently commercialized vaccines have been reported [18,19,20]. Since parenteral administration of vaccines is the most convenient method of vaccine application in the swine industry, strategies like improving delivery techniques [21,22] or the use of molecular adjuvants could be employed to modulate mucosal immunity [23,24].

CC chemokines like CCL27/CTACK and CCL28/MEK secreted from skin keratinocytes and columnar epithelial cells, respectively, have been demonstrated to be capable of enhancing the recruitment of CCR10+ cells and supporting CCR10 expression leukocytes to migrate from the local immunization region to mucosal sites [25,26,27,28,29,30]. On the other hand, the CCL25/TECK expressed by mucosal epithelial cells can chemo-attract CCR9+ cells and support CCR9 expression leukocytes to migrate to the small intestine [30,31]. The application of CCL27 and CCL25 as an adjuvant in DNA vaccines was proven to enhance systemic and/or mucosal immune responses following IM injection in mice or macaques [32,33]. Therefore, the incorporation of these CC chemokines as immune trafficking signals could be a potential strategy for the development of effective parenteral PEDV vaccine regimens.

In the present study, three porcine CCL proteins, namely, CCL27, CCL28, and CCL25 were constructed and stably expressed in the mammalian cell expression system. Different combinations of these chemokines were intramuscularly co-administrated with inactivated PEDV (iPEDV) viral particles and Freund’s adjuvant in pigs. Immunohistochemical (IHC) staining was performed to detect the infiltration of cognate chemokine receptors, CCR9+ or CCR10+, bearing immune cells, to the sites of immunization. The immunogenicity and protective efficiency of iPEDV adjuvanted with Freund’s adjuvant in different combinations of CC chemokines were evaluated in 5-week-old pigs.

## 2. Materials and Methods

### 2.1. Cell Lines and Viruses

The highly virulent PEDV Pingtung 52 passage 5 (PEDVPT-P5) (GenBank Accession No. KY929405) viral stock was passaged for generation of the PEDVPT passage 6 (PEDVPT-P6) and PEDVPT passage 7 (PEDVPT-P7) in Vero C1008 cells (American Type Culture Collection, ATCC No. CRL-1586) as described previously [34,35]. Viral stocks composed of PEDVPT-P6 and PEDVPT-P7 supernatants were in the proportion of 1:1 and used for the animal challenge. The high passaged virulent PEDV Pingtung 52 passage 100 viral stock, namely iPEDV, was filtered through a 0.22 μm pore size membrane and centrifuged at 24,500 rpm for 2.5 h at 4 °C with 20% buffered sucrose. The iPEDV pellets were resuspended in 1 mL DPBS (Gibco), inactivated twice by ultraviolet rays for 20 min, confirmed for no infectivity in Vero cells, and prepared as vaccine immunogens.

### 2.2. Generation of CCL Proteins (CCL27, CCL28, and CCL25)

#### 2.2.1. Plasmid Construction

**Figure 1:**
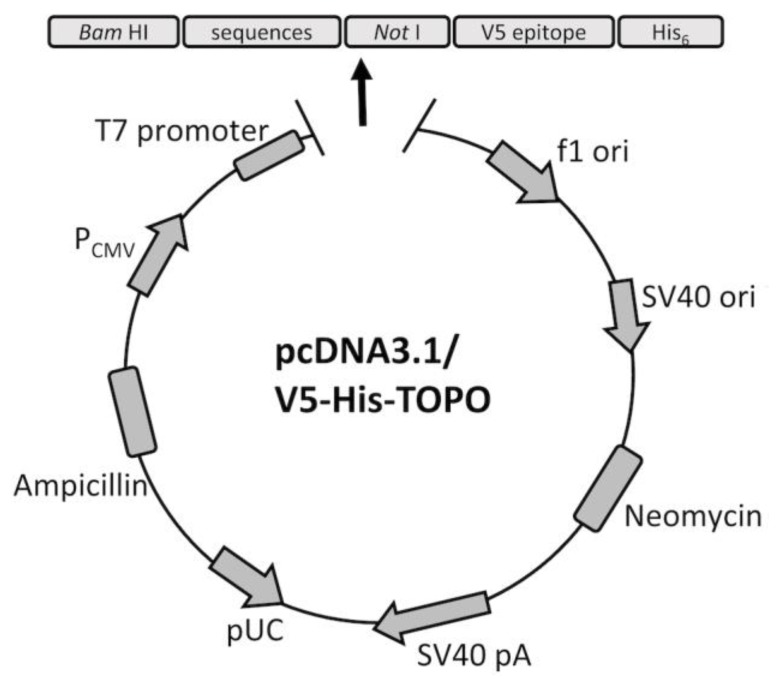
The construction diagram of the porcine CC chemokine proteins. The vector, pcDNA3.1/V5-His-TOPO, was composed of a human cytomegalovirus (CMV) promoter (PCMV), ampicillin and neomycin (G418) resistance genes, pUC-derived origin, a SV40 promoter, f1 origin, and the bovine growth hormone (BGH) polyadenylation signal, including a T7 promoter for in vitro transcription. The porcine CCL27, CCL28, or CCL25 sequences were cloned between restriction enzymes, BamHI and NotI. The V5 epitope and C-terminal polyhistidine tag (His 6) were included for purification of the recombinant proteins.

The nucleotide sequences of CCL27, CCL28, and CCL25 were derived from Sus scrofa C-C motif chemokine ligand 27 (Genbank accession No. NM_001003922), ligand 28 (Genbank accession No. NM_001024695.1), and ligand 25 (Genbank accession No. NM_001025214.1), optimized based on the mammalian expression system and synthesized by the Genscript Corporation (Piscataway, NJ, USA). The synthetic genes were digested with BamHI-NotI restriction sites and cloned into the pcDNA™3.1/V5-His TOPO^®^ vector (Invitrogen, Thermo Fisher Scientific, Waltham, MA, USA) following the manufacturer’s instructions. After transformation in One Shot TOP10 chemically Competent *E. coli* (Invitrogen) following the manufacturer’s guidelines, the three plasmids, namely pcDNA™3.1/CC27/V5-His, pcDNA™3.1/CC28/V5-His, and pcDNA™3.1/CC25/V5-His, were extracted using QIAprep^®^ Spin Miniprep Kit (Qiagen, Hilden, Germany). The clones were confirmed by nucleotide sequencing (Tri-I Biotech Inc., Taipei, Taiwan). The construction diagram is depicted in Figure 1.

#### 2.2.2. Transfections

The pcDNA™3.1/CC27/V5-His, pcDNA™3.1/CC28/V5-His, and pcDNA™3.1/CC25/V5-His plasmids were transfected into human embryonic kidney (HEK) 293 cells (ATCC^®^ CRL-1573™) using PolyJetTM in vitro DNA transfection reagent (SignaGen^®^ Laboratories, MD, USA) following the manufacturer’s recommendations. On the next day, culture medium was replaced by the selective culture medium containing 750 μg/mL Geneticin (G418, Gibco, Thermo Fisher Scientific). Transfected HEK cells were grown in fresh, selective culture medium that was replaced weekly for three weeks to allow generation of cell lines stably expressing porcine proteins CCL27, CCL28, and CCL25. Stable cell lines were further confirmed by immunocytochemistry (ICC) with anti-V5 antibodies (Invitrogen).

#### 2.2.3. Immunocytochemistry (ICC)

HEK 293 cells transfected with CCL27, CCL28, or CCL25 were fixed with 20% ice-cold acetone, and air-dried for one day. Fixed cells were incubated with anti-V5 antibodies (Invitrogen) as primary antibodies at RT for 1 h, followed by monoclonal anti-rabbit/mouse immunoglobulins conjugated with horseradish peroxidase (HRP) (Dako, Carpinteria, CA, USA) as secondary antibodies at RT for another 1 h. The detailed procedure has been described previously [35]. After washing three times with Dulbecco’s Phosphate-Buffered Saline (DPBS) (Gibco), coloration was detected using the EnVision-DAB+ system (Dako, Santa Clara, CA, USA). Positive brown signals were identified under an inverted light microscope (Nikon, Tokyo, Japan).

### 2.3. Purification of CCL (CCL27, CCL28, and CCL25) Proteins

The established HEK 293 cells stably expressing porcine CCL proteins were harvested and resuspended in Gibco^®^ FreeStyle^TM^ 293 expression medium (Invitrogen), and incubated in the CELLSPIN system (INTERGRA bioscience, NH, USA) for three to four days. Supernatant was collected, centrifuged at 3000 rpm for 20 min, filtered through a 0.22 μm pore size membrane, and purified using HisPur cobalt resin (Invitrogen) with Econo-Column^®^ chromatography (Bio-Rad laboratories, Hercules, CA, USA) following the manufacturer’s protocol. The eluted proteins were concentrated using Amicon^®^ Ultra-15 (molecular weight cut-off 3 kDa, Millipore Corp., Bedford, MA, USA), mixed with cOmplete™ EDTA-free protease inhibitor cocktail (Roche Molecular Biochemicals, Laval, QC, Canada), and kept at −20 °C for further experiments.

### 2.4. Western Blotting

Purified CCL proteins were run on a 10% SDS-PAGE gel (HR gradient gel solution, TOOLS, Taipei, Taiwan), and transferred to Immun-Blot^®^ PVDF membranes (Bio-Rad laboratories). The membranes were incubated with anti-V5 monoclonal antibody at 1:5000 dilution (Invitrogen) and then with peroxidase-affiniPure goat anti-mouse IgG (H+L) at 1:10,000 dilution (Jackson ImmunoResearch Laboratories, Inc., PA, USA) as a secondary antibody. The PVDF membranes were developed by the Clarity™ Western ECL blotting substrates (Bio-Rad) and visualized by ChemiDoc™ Imaging Systems (Bio-Rad).

### 2.5. Immunization Program of Piglets

Twenty-six 5-week-old, Large × Duroc, crossbred piglets with no history of PEDV infection were used in this study. The piglets were confirmed to be PEDV-seronegative and PEDV-shedding negative. These pigs were separated into six groups, namely mock group (*n* = 4), iPEDV group (*n* = 4), iPEDV + CCL28 group (*n* = 3), iPEDV + CCL25/28 group (*n* = 5), iPEDV + CCL25/27 group (*n* = 5), and iPEDV + CCL25/27/28 group (*n* = 5). The iPEDV + CCL25 alone and iPEDV + CCL27 alone groups were excluded in the present study owing to the failure of inducing fecal PEDV-specific IgA in our pilot study (Supplementary Figure S1). All pigs were intramuscularly primed with DPBS (Gibco) or 200 μg iPEDV in combination with/without a total of 60 μg of various chemokines. The chemokines were quantified by the bicinchoninic acid (BCA) method using PierceTM BCA protein assay kit (Thermo) and admixed with 0.5 mg of complete Freund’s adjuvant (Sigma-Aldrich, St. Louis, MO, USA), as listed in Table 1. The pigs were boosted with the same formula of immunogens and/or chemokines with 0.5 mg of incomplete Freund’s adjuvant (Sigma-Aldrich) at two weeks post first immunization. Two weeks after the second immunization, pigs were orally challenged with 5 mL of 10^5^ TCID50/mL PEDVPT-P6 and 7 (Table 1). The stool consistency was carefully monitored and recorded daily after the challenge [35]. Ethylenediamine tetraacetic acid (EDTA)-anticoagulated plasma samples, fecal and oral swabs were collected from pigs at the time of the first and second immunization, two weeks after the second immunization, and two weeks after the challenge at 5, 7, 9, and 11 weeks of age for evaluating PEDV-specific systemic IgG and mucosal IgA antibody titers. In addition, fecal swabs were collected daily post-challenge for monitoring fecal PEDV viral shedding. The animal experimental procedure was reviewed and approved by the Institutional Animal Care and Use Committee (IACUC) of the National Taiwan University (Taipei, Taiwan) with approval No. NTU107-EL-00105.

### 2.6. Clinical Signs and Body Weights

Fecal conditions were recorded every day and scored at four degrees: 0, normal; 1, loose; 2, semi-fluid; and 3, watery diarrhea. Body weights of piglets were measured weekly.

### 2.7. Detection of Systemic IgG and Mucosal IgA

To evaluate the levels of PEDV-specific plasma IgG and fecal IgA, we performed an in-house recombinant PEDV S protein-based indirect enzyme-linked immunosorbent assay (ELISA), as previously described [9,35]. The MicroWell^TM^ 96-well microplates (Nunc, Rochester, NY, USA) were coated with 2 μg/mL purified recombinant PEDV S protein and incubated overnight at 4 °C. The microplates were washed six times with 300 μL washing buffer (Kirkegaard and Perry Laboratories (KPL), Milford, MA, USA) by a microplate washer (BioTek Instruments, Inc., Winooski, Vermont, USA) and blocked with blocking buffer (KPL) at RT for 1 h. For detection of plasma IgG, plasma samples at 40-fold dilution in blocking buffer (KPL) were incubated at RT for 1 h; for fecal IgA, fecal suspensions at 2-fold dilution in blocking buffer (KPL) were placed overnight at 4 °C. After six washes, 100 μL of horseradish peroxidase (HRP)-conjugated goat anti-pig IgG (KPL) at a dilution of 1:1000 or HRP-conjugated goat anti-pig IgA (Abcam, Cambridge, UK) at a dilution of 1:5000 were added to detect the levels of porcine IgG and IgA, respectively. After incubation at RT for 1 h and DPBS (Gibco) wash, 50 μL of an ABTS^®^ Peroxidase Substrate System (KPL) was used as a color-developing agent at RT for 5 min (IgG detection) and 20 min (IgA detection), respectively. The color reactions were stopped by adding 50 μL of stopping solution (KPL). The optical density (OD) at 405 nm was determined by an EMax Plus Microplate Reader (Molecular Devices, Crawley, UK).

### 2.8. Detection of Fecal PEDV Viral Loads

To quantitate PEDV viral RNA loads, rectal swabs were resuspended in 900 μL of DPBS (Gibco) with admixture for 5 s and centrifuged at 13,000 rpm for 10 min. The procedures of RNA extraction and cDNA reverse-transcription using the Cador Pathogen 96 QIAcube HT Kit (Qiagen) and QuantiNova Reverse Transcription Kit (Qiagen), respectively, were carried out according to manufacturer’s instructions. The real-time PCR was performed according to our established protocol [9] using the PEDV-N forward primer (3′-CGCAAAGACTGAACCCACTAAC-5′), PEDV-N reverse primer (3′-TTGCCTCTGTTGTTACTTGGAGAT-5′), and PEDV specific probe (3′-FAM-TGYYACCAYYACCACGACTCCTGC-BHQ1-5′). The cycle conditions were as follows: initial denaturation was at 95 °C for 2 min, followed by 45 cycles at 95 °C for 15 s and 55 °C for 15 s.

### 2.9. Viral Neutralization (VN) Assay

The plasma samples of piglets were first incubated at 56 °C for 30 min to inactivate the complement. The neutralizing assay was performed in accordance with the procedure which was previously published [9,35]. The plasma samples were diluted from 10-fold to 320-fold by freshly prepared post-inoculation (PI) medium containing DMEM (Gibco, Gaithersburg, MD, USA) supplemented with 0.3% tryptose phosphate broth (Sigma, St. Louis, MO, USA), 0.02% yeast extract (Acumedia, Lansing, CA, USA), and 10 µg/mL of trypsin (Gibco). The mixture of PEDVPT-P6 and 7 (200 TCID50/mL) and diluted plasma samples at equal volume were added to each well and incubated at 37 °C for 1 h and applied to Vero cell-coated microplates for another hour of incubation. Then, these microplates were washed and maintained in PI medium for 24 h. The cytopathic effect (CPE) was identified under an inverted light microscopy (Nikon). The neutralizing titers of the plasma samples were determined as the highest dilution without CPE.

### 2.10. In Vivo Functional Assay of CCL Proteins (CCL27, CCL28, and CCL25)

Fifteen 5-week-old, Large × Duroc, crossbred piglets selected from a conventional pig farm with no history of PEDV infection were used to confirm the bio-function of the above immunization program of piglets, which showed protection against the highly virulent PEDV challenge. These pigs were randomly separated into five groups, including the mock group (*n* = 3), the iPEDV group (*n* = 3), the iPEDV + CCL25/28 group (*n* = 3), the iPEDV + CCL25/27 group (*n* = 3), and the iPEDV + CCL25/27/28 group (*n* = 3). All 5-week-old pigs received two IM injections into the biceps femoris muscles of their left leg, and primed and boosted with the same immunization program at 5 and 7 weeks old, as previously described (Table 1). The injected biceps femoris muscles were excised at one week following the second immunization, fixed in 10% neutral-buffered formalin, and paraffin-embedded muscles were serially sectioned into sections 5 μm thin, stained with hematoxylin and eosin for examining the existence of locally extensive inflammatory infiltrates, and then analyzed by immunohistochemistry (IHC) to visualize inflammatory cells expressing their specific receptors, CCR9 (the receptor of CCL25) and CCR10 (the receptor of both CCL27 and CCL28). The animal experimental procedure was reviewed and approved by the Institutional Animal Care and Use Committee (IACUC) of the National Taiwan University (Taipei, Taiwan) with approval No. NTU107-EL-00105.

### 2.11. Immunohistochemistry (IHC)

The paraffin-embedded tissue sections were de-paraffinized with xylene, rehydrated with serially descending ethanol gradient, and rinsed with Tris-buffered saline containing 0.1% Tween 20 (TBST). The antigens were retrieved by the proteinase K solution (Qiagen) diluted 1:200 in phosphate-buffered saline (PBS; pH 7.4) at RT for 15 min. After three washings by TBST, the slides were blocked with 10% normal goat serum (Dako) at RT for 30 min, then separately incubated with CCR9 polyclonal antibodies (1 mg/mL, diluted 1:250 in PBS) (Invitrogen) or CCR10 polyclonal antibodies (1 mg/mL, diluted 1:200 in PBS) (Invitrogen) at RT for an hour. Endogenous peroxidases were inactivated by 3% hydrogen peroxide in methanol at RT for 15 min. The slides were rinsed with TBST, stained by the EnVision-HRP system (rabbit/mouse) (Dako Denmark A/S, Glostrup, Denmark) at RT for an hour, and visualized by using the EnVision-DAB+ system (Dako) with Mayer’s hematoxylin solution (MUTO, Tokyo, Japan) counterstained. The tissue section stained synchronously without applying the primary antibodies was used as a negative control. Positive brown signals identified in the cytoplasm or membranes of injection-induced inflammatory cells were further evaluated by visual counting of 10 independent, randomly selected 200× high power fields from each group in a blind pattern by two individual examiners, repeated twice [33].

### 2.12. Statistical Analysis

The results of body weights, antibody titers, and viral shedding were statistically calculated by the Statistical Analysis System (SAS, SAS Institute Inc., Cary, NC, USA) with one-way ANOVA, following reciprocal transformation due to the test of additivity, test of independence of error, and test of homogeneity of variance. As to the results of immunohistochemistry between each immunization group, they were calculated by SAS with a two-tailed, paired Student’s *t* test. Statistical differences were considered significant with a *p*-value < 0.05.

## 3. Results

### 3.1. Expression and Detection of Porcine CCL25, CCL27, and CCL28

**Figure 2:**
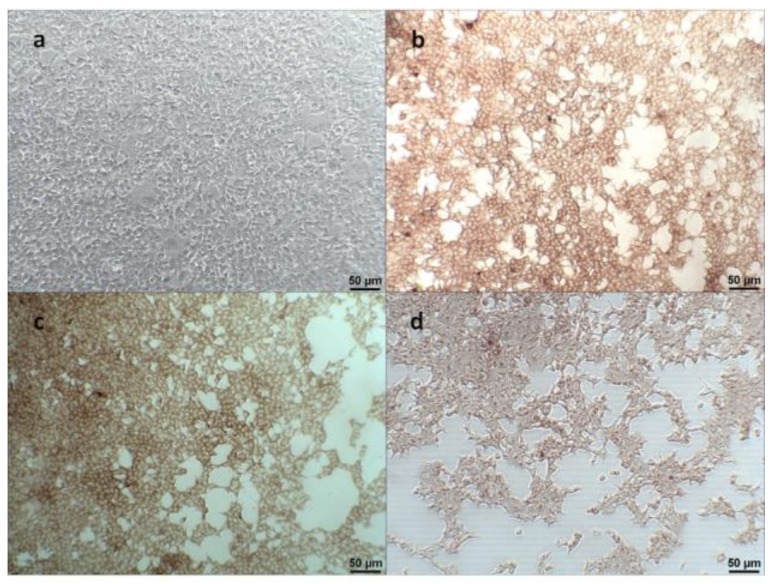
(**a**) Detection of the expression of CC chemokines by immunocytochemistry (ICC) in the naïve parental HEK-293 cells, (**b**) HEK-293 cell lines stably expressing porcine CCL27, (**c**) CCL28, and (**d**) CCL25. The anti-V5 antibodies (1:1000 dilution) was used as the primary antibody, and the polyclonal anti-rabbit/mouse immunoglobulins conjugated with horseradish peroxidase (HRP) was used as the secondary antibody. The coloration was detected using the EnVision-DAB+ system (Dako, Santa Clara, CA, USA). Positive brown signals were identified under an inverted light microscope (Nikon, Tokyo, Japan).

**Figure 3:**
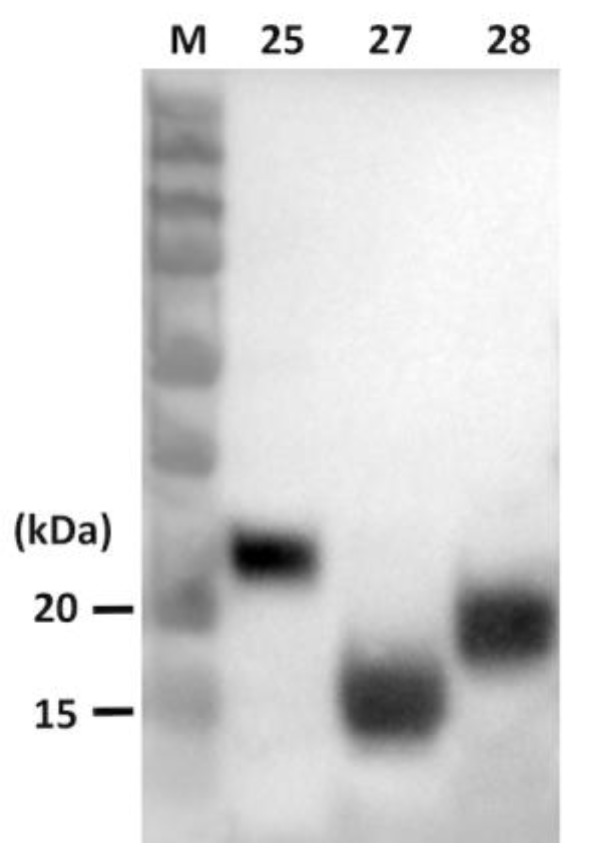
Western blotting analysis of the expression of porcine CCL25 (lane 25), CCL27 (lane 27), and CCL28 (lane 28) proteins. The recombinant CC chemokine proteins were detected by anti-V5 antibodies (1:5000 dilution) as the primary antibody and alkaline peroxidase conjugated goat anti-mouse IgG (1:10,000 dilution) as the secondary antibody. The polyvinylidene difluoride (PVDF) membranes were developed by the Clarity™ Western enhanced chemiluminescence (ECL) blotting substrates (Bio-Rad) and visualized by ChemiDoc™ Imaging Systems (Bio-Rad).

For the expression of porcine proteins, HEK293 cell lines were cultivated in a selection medium containing G418. After 3–4 weeks, HEK293 cell lines stably expressing CCL27, CCL28, and CCL25 were successfully generated. To characterize and determine the expression levels of CCL proteins, immunocytochemistry (ICC) and western blotting using anti-V5 antibodies (Invitrogen) were performed. While no detectable signal was noted in normal HEK 293 cells, more than 90% of the three established chemokine-expressing cells exhibited intracytoplasmic positive signals (Figure 2). Using the western blotting analysis, the molecular weights of the porcine proteins CCL25, CCL27, and CCL28 were determined to be about 25, 15, and 17 kilodaltons (kDa), consistent with the predicted sizes of 21, 15, and 18 kDa, respectively (Figure 3).

### 3.2. Detection of PEDV-Specific Systemic IgG and Mucosal IgA by ELISA

**Figure 4:**
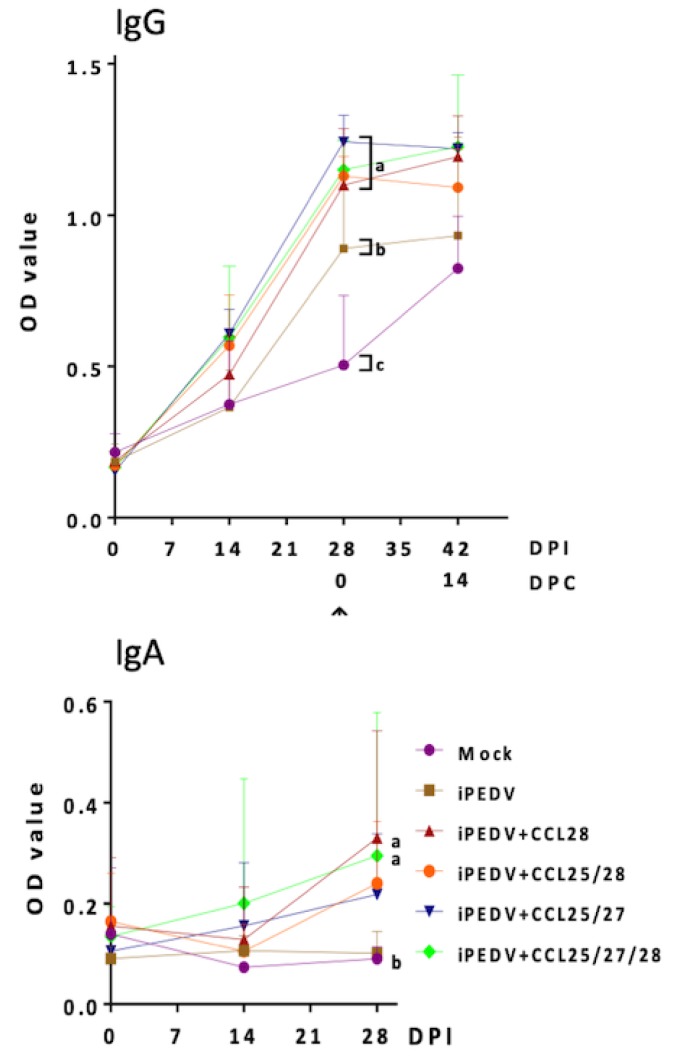
The detection of systemic PEDV spike (S)-specific IgG and fecal IgA in piglets. The PEDV-specific IgG in blood was detected at 0, 14, and 28 days post-immunization (DPI) and at 14 days post-challenge (DPC) following the challenge with the virulent PEDV, PEDVPT-P6 and 7. The mucosal PEDV-specific fecal IgA was detected in piglets at 0, 14, and 28 DPI. Data is displayed as the average optical density (OD) values of each group with error bars representing the standard deviation (SD). The arrow reflects the particular time (28 DPI or 0 DPC) of the PEDVPT-P6 and 7 challenge. Statistically significant differences are present among a, b, and c (*p* < 0.05).

**Figure 5:**
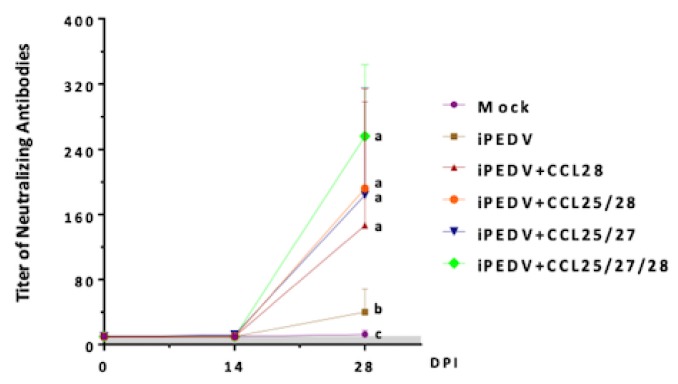
The titers of viral neutralizing (VN) antibodies against PEDV in each group at 0, 14, and 28 days post-immunization (DPI). Data are presented as the mean titer ± standard deviation. The gray zone is the background of the viral neutralizing assay. Statistically significant differences are present among a, b, and c (*p* < 0.05).

To evaluate whether co-administration of porcine CCL25, 27, and/or 28 could modulate systemic and mucosal immune responses, IM vaccination regimens using iPEDV as the immunogen with/without different combinations of porcine CCL25, 27, and/or 28 proteins and Freund’s adjuvant were compared. As compared with the mock group, the iPEDV group elicited systemic PEDV-specific IgG (Figure 4) and VN antibody titers (Figure 5) but failed to elicit a detectable PEDV-specific fecal IgA response (Figure 4). Interestingly, pigs of all chemokine-adjuvanted groups not only induced superior PEDV-specific IgG levels (Figure 4) and viral neutralizing (VN) antibody titers (Figure 5) as compared to those of mock and iPEDV groups, but also induced variable and detectable levels of PEDV-specific fecal IgA (Figure 4). Statistically significant higher levels of PEDV-specific IgA were detected in the iPEDV+CCL28 and iPEDV+CCL25/27/28 groups as compared to the mock group at 28 days post-immunization (DPI) (Figure 4).

### 3.3. Enhancement of Protective Efficacy of CCL25, 27, and/or 28 Adjuvanted iPEDV Vaccines against the Virulent PEDVPT-P6 and 7 Challenge

**Figure 6:**
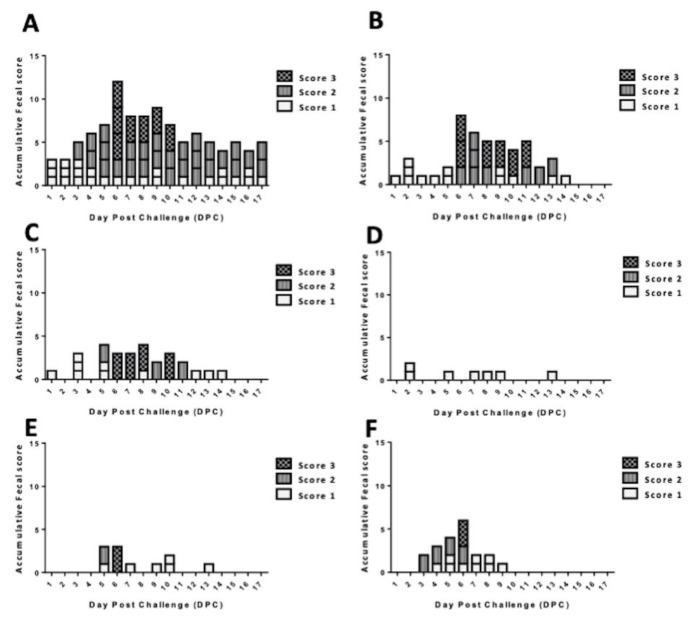
The accumulative scores of fecal conditions in (**A**) the mock group, (**B**) the iPEDV group, (**C**) the iPEDV + CCL28 group, (**D**) the iPEDV + CCL25/28 group, (**E**) the iPEDV + CCL25/27 group, and (**F**) the iPEDV + CCL25/27/28 group. The clinical signs of feces are classified into four grades: score 0, normal; score 1, loose; score 2, semi-fluid; and score 3, watery diarrhea.

To evaluate the protective efficacy, pigs receiving IM vaccination of iPEDV as an immunogen with/without different combinations of porcine CCL25, 27, and/or 28 proteins combined with Freund’s adjuvant were challenged with the virulent PEDVPT-P6 and 7 at 28 DPI. The clinical signs, fecal conditions, daily viral shedding, weekly body weights, and VN antibody titers were evaluated. During the study, no statistically significant difference in weekly body weights was observed among different groups (data not shown). Regarding the fecal conditions, no clinical signs were observed in the pigs before the PEDV challenge. After oral inoculation with PEDVPT-P6 and 7, all pigs in the mock and iPEDV group showed typical PEDV-associated diarrhea, in which moderate to severe watery stools (score 2–3) were noted in 4 out of 5 and 3 out of 4 pigs at 3–17 and 6–13 days post-challenge (DPC), respectively (Figure 6A,B). In the iPEDV + CCL28 group, although all three pigs exhibited clinical diarrhea, only 1 out of 3 piglets showed moderate to severe watery stools (score 2–3) during 5–11 DPC (Figure 6C). Conversely, pigs in both iPEDV + CCL25/27 and iPEDV + CCL25/27/28 groups had a relatively shortened course of severe symptoms, showing moderate to severe watery stools (score 2–3) during 5–6 and 3–6 DPC, respectively (Figure 6E,F). In pigs immunized with iPEDV adjuvanted with both CCL25 and CCL28, only intermittent loose diarrhea (score 1) was detected in 2 out of 5 pigs during the PEDV challenge (Figure 6D). Importantly, no severe diarrhea (score 2–3) was noted in pigs of this group.

### 3.4. Viral Shedding of Highly Virulent PEDVPT- P6 and 7 Challenge Discovered by a Probe-Based Quantitative Reverse Transcription PCR (RT-qPCR) Targeting on the N Gene of PEDV

**Figure 7:**
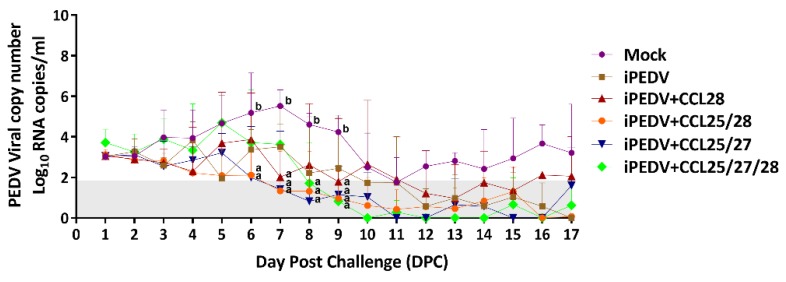
The average mean values of PEDV viral copy numbers detected in the feces of piglets following the virulent PEDVPT-P6 and 7 challenge. The fecal PEDV viral load was detected by a probe-based quantitative reverse transcription PCR (RT-qPCR) and transformed into the log10 copies/mL. The error bars indicate the standard deviation. The limit of detection for RT-qPCR was 1.8 log10 (copies/mL) marked as the gray zone. Statistically significant differences are present between a and b (*p* < 0.05).

The level of shedding of PEDV in each group during 1–17 DPC is depicted in Figure 7. In the mock group, the average viral shedding titer was 3.02 ± 0.13 log10 copies/mL at 1 DPC, uninterruptedly climbing and fluctuating at 3–9 DPC with a peak viral titer of 5.52 ± 0.68 log10 copies/mL at 7 DPC, and dropping at 10 DPC. In the iPEDV group, the viral shedding titer was 3.04 ± 0.09 log10 copies/mL at 1 DPC, dramatically fluctuating at 3–7 DPC, and decreasing at 8 DPC. In the iPEDV + CCL28 immunized group, the average viral shedding titer reached the peak of 3.87 ± 1.87 log10 copies/mL, plummeting to 2.01 ± 2.84 log10 copies/mL at 7 DPC. As compared to the mock group, statistically significant lower viral shedding titers were noted at 7 and 9 DPC (*p* < 0.05). In the iPEDV + CCL25/28 group, the average viral shedding titer was 3.12 ± 0.22 log10 copies/mL at 1 DPC with a minor drop to 2.82 ± 0.32 log10 copies/mL at 3 DPC. As compared with the mock group, significantly lower average viral shedding titer was noted during 6–9 DPC (*p* < 0.05). The iPEDV + CCL25/27 group had an average viral shedding titer of 3.05 ± 0.09 log10 copies/mL at 1 DPC, reached a peak viral shedding of 3.21 ± 0.83 log10 copies/mL at 5 DPC, and gradually decreased during 6–8 DPC. As compared to the mock group, statistically significant lower viral shedding titers were noted during 6–9 DPC (*p* < 0.05). In the iPEDV + CCL25/27/28 group, the average viral shedding titer was 3.71 ± 0.57 log10 copies/mL at 1 DPC, fluctuated during 1–6 DPC with a peak viral titer of 4.69 ± 1.32 log10 copies/mL at 5 DPC, and reduced after 7 DPC with a statistically significant difference (*p* < 0.05) when compared to the mock group at 8–9 DPC. After 9 DPC, no statistically significant difference in the average viral shedding titer was noted among the six groups.

### 3.5. Evaluation of In Vivo Functional Assay of CCL25, 27, and/or 28 Attracting CCR9+ and/or CCR10+ Inflammatory Cells Surrounding Injection-Site Regions

**Figure 8:**
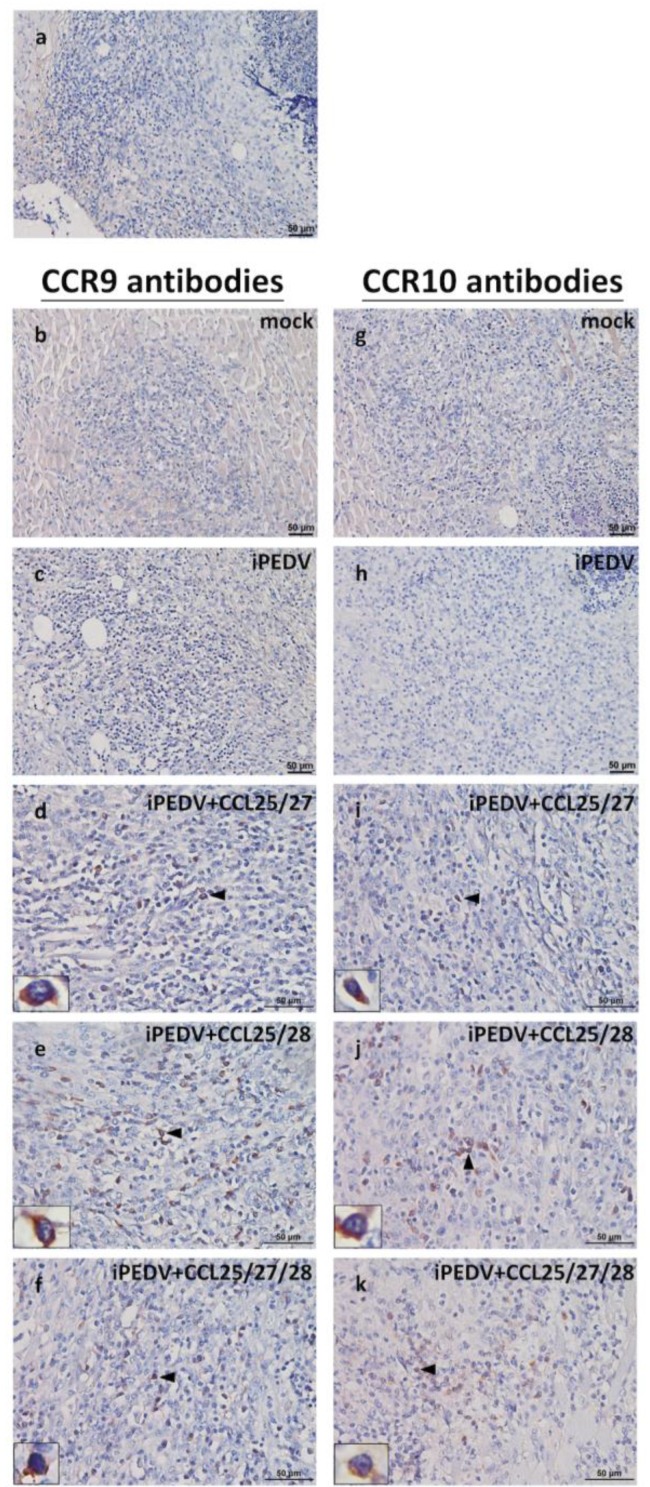
Detection of the expression of CCR9 and CCR10 antigens in injection-site inflammatory cells by immunohistochemistry (IHC). Those infiltrating inflammatory cells were incubated with an anti-CCR9 and CCR10 antibodies (1:250 and 1:200 dilution) as the primary antibody, conjugated with the EnVision-HRP system (rabbit/mouse) as the secondary antibody, and then visualized by the EnVision-DAB+ system by a light microscopy. (**a**) The tissue section stained synchronously without applying the primary antibodies was taken as a negative control. (**d**,**i**) Scattering CCR9 and CCR10 antigens were observed in the cytoplasm of inflammatory cells in the iPEDV + CCL25/27, (**e**,**j**) iPEDV + CCL25/28, and (**f**,**k**) iPEDV + CCL25/27/28 group. Rare CCR9 and CCR10 positive signals were detected in the mock (**b**,**g**) and iPEDV (**c**,**h**) group. Scale bars are 50 μm.

**Figure 9:**
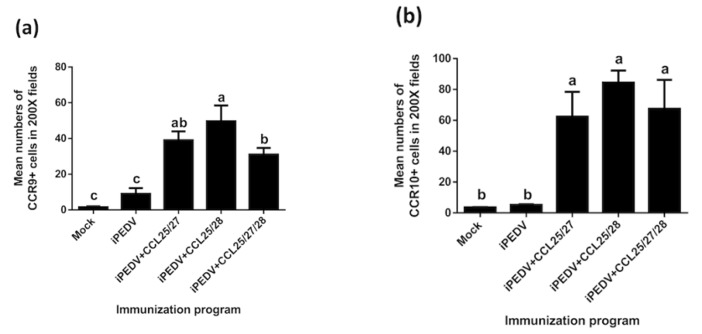
The average numbers of (**a**) CCR9 and (**b**) CCR10 positive cells were counted by a light microscopy over 10 different (200×) fields in each group at one week post the second immunization. Data are presented as the mean number ± standard deviation. Statistically significant differences are present among a, b, and c (*p* < 0.05).

As compared to pigs vaccinated with or without iPEDV alone, pigs vaccinated with iPEDV in combination with quantitative and purified CCL proteins (CCL25, 27, and/or 28) showed variable immune protection against the virulent PEDV challenge. To verify the in vivo function of CCL proteins consistent with the immune protection in each group of the PEDV-challenged animal, we repeated the same immune formula to disclose the in vivo immunogenicity of CC chemokines. Distinct CCR9+ (Figure 8b–f) and/or CCR10+ (Figure 8g–k) antigens were scattered in inflammatory cells at the injection site in varying degrees among immunization groups. Those inflammatory cells were further separately quantitated, as shown in Figure 9a,b. In the detection of CCR9 antigens, mean numbers of CCR9+ cells in the iPEDV + CCL25/27, iPEDV + CCL25/28, and iPEDV + CCL25/27/28 groups (39 ± 3.5, 49.65 ± 6.25, and 31 ± 2.6, respectively) shared statistically significant differences (*p* < 0.01) as compared to both the mock and iPEDV groups (0.4 ± 1.5 and 9 ± 2.3, respectively) (Figure 9a). Notably, the values of CCR9+ cells in the iPEDV + CCL25/28 group also showed statistical differences (*p* < 0.05) compared to the iPEDV + CCL25/27/28 group (Figure 9a). On the other hand, the data in the detection of CCR10 antigens mirrored that of CCR9 antigens. Mean numbers of CCR10+ inflammatory cells in the iPEDV + CCL25/27, iPEDV + CCL25/28, and iPEDV + CCL25/27/28 groups (62.3 ± 11.4, 84.35 ± 5.55, and 67.45 ± 13.25, respectively) shared statistically significant differences (*p* < 0.01) in comparison to the mock and iPEDV groups (3.5 ± 0.2 and 5.2 ± 0.3, respectively) (Figure 9b).

## 4. Discussion

The stimulation of mucosal immunity by trafficking antigen secreting cells (ASCs) to mucosa-related locations has been considered difficult via IM administration of vaccinations. However, IM administration is the most popular and convenient route for vaccinations in pig herds [6,15,16,17,36,37]. In the present study, we have demonstrated that IM co-administration of iPEDV with CC chemokines as adjuvants could induce superior systemic antigen-specific IgG, mucosal antigen-specific IgA, and recruitment of CCR9+ and/or CCR10+ inflammatory cells at the injection site of the experimental pigs as compared to control piglets. Strikingly, pigs intramuscularly immunized with iPEDV in combination with chemokines CCL25 and CCL28 were almost fully protected from the virulent PEDV challenge. Taken together, our results suggest that the application of porcine CC chemokines as the iPEDV IM vaccine adjuvants could successfully augment vaccine immune responses at the mucosal sites.

Chemokines such as CCL25, CCL28, and CCL27 secreted from the vascular endothelial cells or skin keratinocytes play a critical role in homing lymphocytes and/or ASCs by encoding their corresponding receptors at mucosal sites of pathogenic exposure [18,27,28,37]. Both CCL27 and CCL28 have been demonstrated as being able to recruit CLA- positive cells expressing the cognate receptor CCR10 and its ligands in the chemo-trafficking of antigen-specific IgA secreting plasmablasts and memory CD4+ T lymphocytes to mucosal sites of pathogenic exposure [25,28,38,39,40]. These functions had been applied in the development of vaccines for HIV-1 DNA and virus-like particles (VLP). In the HIV-1 DNA vaccine, IM co-administration of HIV antigens adjuvanted with CCL27 plasmid in both mice and macaques has been demonstrated to successfully magnify the HIV-specific IgA titers at mucosal areas [32]. In the case of HIV-1 VLPs co-immunized with CCL28 plasmid, HIV-specific IgA, as well as gastro-intestinal mucosal IgA-secreting plasma cells were significantly augmented in mice [41]. On the other hand, co-administration of CCL25 plasmid with the HIV DNA vaccine showed the elevation of both systemic HIV-specific IgG and mucosal HIV-specific IgA immune responses in mice following IM immunization [33].

In pigs, similar abilities to recruit CCR9+ or CCR10+ cells and facilitate them homing to the mucosal sites have been demonstrated in porcine CCL25, 27 or 28 [18,26,30,37,42]. Differing from previous studies in using plasmid of CC chemokines as mucosal adjuvants in DNA vaccines [32,33,43], purified mammalian cells-derived porcine chemokines were used instead in the present study. While the level of protein expression and immunogenicity of chemokine plasmids might be variable in different individuals, mammalian cell-derived porcine chemokines proteins used in the present study could provide a standardized and quantitative examination. To our knowledge, this is the first study demonstrating that the porcine chemokine proteins can be IM co-administrated and capable of modulating mucosal immunity in pigs. As compared to pigs vaccinated with iPEDV in combination with CCL25/27 and CCL25/28, pigs vaccinated with iPEDV in combination with CCL28 alone or CCL25, CCL27, and CCL28 showed minimal protection against the virulent PEDV challenge. Concerning in vivo functions of CC chemokines, the findings unveiled that the iPEDV + CCL25/28 group also showed superior in vivo chemotaxis of CCR9+ cells compared to the iPEDV + CCL25/27/28 group. These results may help to explain that the co-existence of CCL25 and CCR9+ cells attracting chemokines is necessary for the IM iPEDV vaccine to mount effective immune protection against PEDV. Furthermore, the less effective CCL25, CCL27, and CCL28 triple-adjuvanted iPEDV vaccine, which contained 20 μg of each chemokine per shot, suggested that the amount of each chemokine should be at least 30 μg per shot to reach similar efficacy of disease protection, such as in the CCL25/CCL28 or CCL25/CCL27 double-adjuvanted groups.

While all groups with IM co-administration of iPEDV adjuvanted with different combinations of CC chemokines could induce superior systemic antigen-specific IgG and mucosal antigen-specific IgA responses as compared to those without CC chemokines and control piglets, only pigs in groups vaccinated with iPEDV in combination with both CCL25 and CCL28, or both CCL25 and CCL27 showed significant reduction in viral shedding and clinical signs after the virulent PEDV challenge. Co-administration of both CCL25 and CCL28, or both CCL25 and CCL27 has been demonstrated not only to recruit and home CCR9+ and CCR10+ cells to mucosal sites, but also enhance mucosal antigen-specific CD8+ T cells and the secretion of IFN-γ, TNF-α and IL-2 [32,33,41,44,45,46,47,48]. It has also been shown that both CD4+ and CD8+ T cell responses and interferons (IFNs) might be critical interfering factors against PEDV [18,37,47,48] besides the antigen-specific IgA. Further investigations for disclosing the exact immune-protective mechanism of CC chemokines-adjuvanted iPEDV vaccines, such as functional assays for determining antibody secreting cells, chemotaxis studies for attracting CCR-expressing cells, and ELISpots for studying the PEDV specific cellular responses in the mucosal site, are necessary.

## 5. Conclusions

In this study, we successfully generated three porcine CCL (CCL27, 28, and 25) proteins based on the stable mammalian cell expression system. The combinations of CCR10 and CCR9 attracting chemokines have been demonstrated to be potential novel mucosal adjuvants for an inactivated G2b PEDV vaccine. This novel invention and combination allows us to develop an IM iPEDV vaccine capable of eliciting PEDV-specific systemic and mucosal immune responses, relieving clinical signs, and reducing viral shedding. Moreover, our porcine CC chemokines might be applied to the development of other porcine vaccines against mucosal transmissible pathogens, such as the porcine deltacoronavirus (PDCoV) and porcine reproductive and respiratory syndrome virus (PRRSV). Future research should concentrate on whether co-immunization with CC chemokines and iPEDV provides lactogenic immunity and similar protective efficacy for suckling piglets against PEDV.

## Figures and Tables

**Table 1:** Design of immunization programs and animal experiment.

Group Name	Antigen	Immunization Program of Adjuvants	
		Freund’s Adjuvant	CCL Adjuvant
Mock	None	1st: 0.5 mg of complete Freund’s adjuvant 2nd: 0.5 mg of incomplete Freund’s adjuvant	None
iPEDV *	200 μg of iPEDV		None
iPEDV * + CCL28			60 μg of CCL28
iPEDV * + CCL25/28			30 μg of each CCL25 and CCL28
iPEDV * + CCL25/27			30 μg of each CCL25 and CCL27
iPEDV * + CCL25/27/28			20 μg of each CCL25, CCL27 and CCL28

* iPEDV: inactivated PEDV.

## Supplementary Materials

The following are available online at https://www.mdpi.com/2076-393X/8/1/102/s1. Figure S1: The detection of systemic PEDV spike (S)-specific IgG and fecal IgA in iPEDV + CCL25 and/or CCL27 immunized piglets.
