# Supplementary material for: Intramuscular Immunization with Chemokine-Adjuvanted Inactive Porcine Epidemic Diarrhea Virus Induces Substantial Protection in Pigs
Source: Vaccines (Basel). 2020 Feb 24;8(1):102. doi: 10.3390/vaccines8010102 (PMC7157555; doi:10.3390/vaccines8010102)
Supplement: Supplementary file 1 [file vaccines-08-00102-s001.pdf]

Supplementary Figure S1

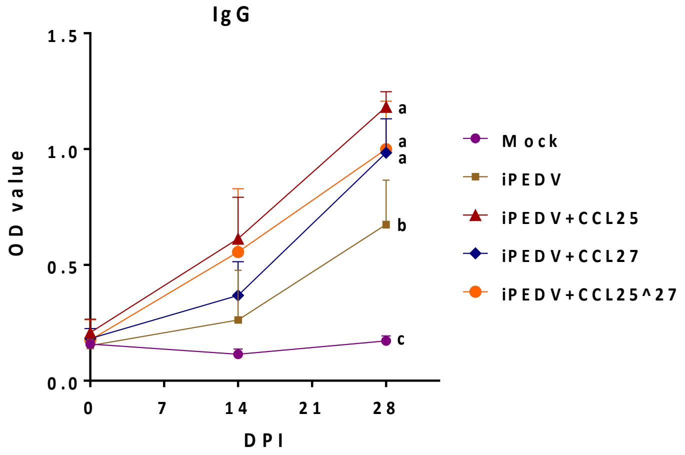

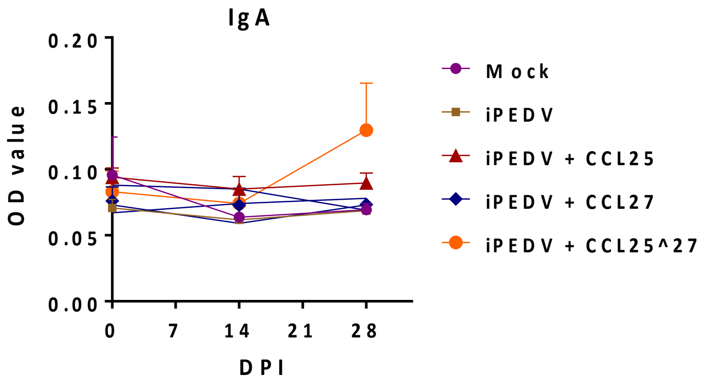

Figure 1. The detection of systemic PEDV spike (S)-specific IgG and fecal IgA in piglets. The PEDV specific IgG in blood was detected at 0, 14, and 28 days post-immunization (DPI). The mucosal PEDV specific fecal IgA was detected in piglets at 0, 14, and 28 DPI. Data is displayed as the average optical density (OD) values of each group with error bars representing the standard deviation (SD). Statistically significant differences are present among a, b and c ($p < 0.05$).
